# Supplementary material for: Developmental Disorder Probability Scores at 6–18 Years Old in Relation to In-Utero/Peripartum Antiretroviral Drug Exposure among Ugandan Children
Source: Int J Environ Res Public Health. 2022 Mar 21;19(6):3725. doi: 10.3390/ijerph19063725 (PMC8955488; doi:10.3390/ijerph19063725)
Supplement: Supplementary file 1 [file ijerph-19-03725-s001.zip › ijerph-1574809-supplementary.pdf]

International Journal of *Environmental Research and Public Health*

MDPI

Article

Developmental Disorder Probability Scores at 6–18 Years Old in Relation to In-Utero/Peripartum Antiretroviral Drug Exposure among Ugandan Children

Supplementary Materials

Table S1. Biological sex, developmental stage, and early-life factors in relationship to ADHD, ASD, and EBD probability indices and overall resiliency among 6–18-year-old children from Uganda.

		ADHD Probability Index		ASD Probability Index		EBD Probability Index		Resiliency Index	
	**n**	**LSM ± SE**	**SMD (95% CI)**	**LSM ± SE**	**SMD (95% CI)**	**LSM ± SE**	**SMD (95% CI)**	**LSM ± SE**	**SMD (95% CI)**
**Child Sex**									
Female	390	−0.18 ± 0.07	−0.12 (−0.28, 0.04)	−0.7 ± 0.06	−0.06 (−0.18, 0.06)	0.14 ± 0.08	**−0.18 (−0.37,−0.07)**	0.23 ± 0.06	0.03 (−0.12, 0.19)
Male	348	−0.06 ± 0.07	Ref	−0.01 ± 0.07	Ref	−0.08 ± 0.07	Ref	0.19 ± 0.07	Ref
Apgar Score at Birth (per unit increase)	n/a	–	−0.06 (−0.15, 0.03)	–	**−0.12 (−0.20, −0.03)**	–	−0.04 (−0.10,0.02)	–	**0.14 (0.05, 0.23)**
Birthweight (per kg increase)	n/a	–	**−0.19 (−0.39, −0.00)**	–	**−0.18 (−0.33,−0.03)**	–	−0.02 (−0.16, 0.12)	–	0.13 (−0.02, 0.27)
**Lifetime Adversity**									
Low	389	−0.03 ± 0.06	**0.22 (0.09, 0.35)**	0.03 ± 0.06	**0.21 (0.09, 0.34)**	0.12 ± 0.06	**0.26 (0.14, 0.38)**	0.12 ± 0.05	−0.06 (−0.19, 0.08)
High	366	−0.25 ± 0.07	Ref	−0.18 ± 0.05	Ref	−0.14 ± 0.06	Ref	0.18 ± 0.06	Ref
**Caregiver Depression**									
Not depressed	596	−0.16 ± 0.05	−0.07 (−0.22, 0.09)	−0.13 ± 0.05	−0.12 (−0.28, 0.04)	−0.12 ± 0.05	**−0.25 (−0.42, −0.08)**	0.10 ± 0.05	−0.10 (−0.24, 0.05)
Depressed	159	−0.09 ± 0.08	Ref	−0.01 ± 0.08	Ref	0.13 ± 0.08	Ref	0.20 ± 0.07	Ref
**Functioning in Caregiving Role**									
Lowest Tertile	186	−0.14 ± 0.08	**0.13 (−0.03, 0.30)**	−0.06 ± 0.07	**0.17 (0.02, 0.32)**	0.09 ± 0.07	**0.35 (0.18, 0.52)**	0.16 ± 0.06	−0.01 (−0.18, 0.16)
Middle Tertile	363	0.05 ± 0.08	**0.32 (0.18, 0.49)**	0.07 ± 0.06	**0.29 (0.14, 0.44)**	0.18 ± 0.07	**0.45 (0.29, 0.60)**	0.11 ± 0.07	−0.07 (−0.22, 0.09)
Highest Tertile	206	−0.28 ± 0.07	Ref	−0.22 ± 0.08	Ref	−0.27 ± 0.07	Ref	0.18 ± 0.07	Ref

Multivariable regression models adjusted for child (age, sex), caregiver (education, lifetime adversity, depressive symptoms, caregiver functioning in caregiving role, social support), and early-life child health indicators (birthweight, APGAR score); n/a = not applicable; bolded associations are statistically significant at alpha = 0.05.

Table S2. HIV treatment factors and select demographic factors in relationship to developmental disorder, resiliency, and functional impairment outcomes at 6–18 years of life among children perinatally infected.

		ADHD Probability Index	EBD Probability Index	ASD Probability Index	Resiliency Index	Functional Impairment Index
	n	SMD (95% CI)	SMD (95% CI)	SMD (95% CI)	SMD (95% CI)	SMD (95% CI)
**CD4 Nadir**						
Lowest (<400, mean = 229)	70	0.09 (−0.21, 0.39)	−0.11 (−0.42, 0.20)	0.15 (−0.14, 0.45)	−0.05 (−0.35, 0.27)	0.13 (−0.19, 0.46)
Medium (400-689, mean = 535.5)	77	0.12 (−0.17, 0.40)	−0.13 (−0.44, 0.19)	**0.39 (0.12, 0.67)**	−0.12 (−0.41, 0.17)	0.21 (−0.10, 0.52)
High (>=690, mean = 1041.52)	71	Ref	Ref	Ref	Ref	Ref
**Age at cART Initiation**						
**>18 months**	74	0.05 (−0.21, 0.33)	**0.29 (0.03, 0.57)**	0.20 (−0.05, 0.45)	0.12 (−0.36, 0.13)	0.07 (−0.21, 0.35)
**≤18 months**	165	Ref	Ref	Ref	Ref	Ref
**Current ART Regimen ****						
NRTI-based regimen	53	−0.20 (−0.48, 0.08)	0.01 (−0.25, 0.27)	−0.17(−0.46, 0.11)	0.19 (−0.09, 0.49)	−0.05 (−0.34, 0.24)
NNRTI-based regimen	60	Ref	Ref	Ref	Ref	Ref
PI-based regimen	122	−0.17 (−0.44, 0.10)	−0.08 (−0.39, 0.23)	−0.18 (−0.48, 0.11)	0.15 (−0.16, 0.46)	−0.21 (−0.51, 0.11)
No known current ART	7	0.23 (−0.37, 0.82)	0.18 (−0.30, 0.66)	**0.47 (0.01, 0.93)**	0.08 (−0.29, 0.45)	**0.58 (0.07, 1.09)**
Lifetime Adversity						
High	53	**0.31 (0.07, 0.54)**	**0.32 (0.09, 0.54)**	**0.29 (0.05, 0.52)**	0.13 (−0.40, 0.14)	**0.45 (0.22, 0.68)**
Low	189	Ref	Ref	Ref	Ref	Ref
**Birth APGAR score**						
Per unit increase	-	−0.04 (−0.15, 0.07)	−0.01 (−0.14, 0.12)	−0.10 (−0.23, 0.02)	**0.14 (0.03, 0.25)**	−0.05 (−0.18, 0.08)
Low Apgar (<=6)	9	**0.67 (0.39, 0.94)**	0.41 (−0.06, 0.89)	**0.46 (0.11, 0.81)**	**−0.50 (−0.90, −0.11)**	**0.31 (0.01, 0.61)**
**High Apgar (>=7)**	232	Ref	Ref	Ref	**Ref**	Ref
**Birth weight**						
Per Kg increase	-	−0.11 (−0.32, 0.10)	−0.04 (−0.29, 0.22)	−0.05 (−0.28, 0.18)	0.20 (−0.03, 0.43)	−0.13 (−0.36, 0.10)
LBW (<2500 kg)	16	0.08 (−0.33, 0.49)	−0.16 (−0.66, 0.34)	0.20 (−0.16, 0.57)	−0.34 (−0.76, 0.08)	0.07 (−0.41, 0.54)
Normal (>=2500 kg)	220	Ref	Ref	Ref	Ref	Ref
**Caregiver functioning**						
Low		0.23 (−0.05, 0.51)	**0.58 (0.29, 0.86)**	**0.35 (0.07, 0.62)**	−0.13 (−0.43, 0.18)	**0.55 (0.24, 0.86)**
Moderate		**0.29 (0.03, 0.56)**	**0.53 (0.27, 0.79)**	**0.31 (0.04, 0.57)**	−0.17 (−0.44. 0.10)	**0.42 (0.15, 0.68)**
High		**Ref**	**Ref**	Ref	Ref	**Ref**

Regression models are sequentially adjusted for respective HIV-related factors: viral suppression, timing of ART initiation, current ART regimen. ** PI includes Ritonavir, Atazanivar, or Lopinavir/Kaletra; NNRTI = EFV or NVP; NRTI = Abacavir or TDF inclusive. Bolded numbers are statistically significant associations.

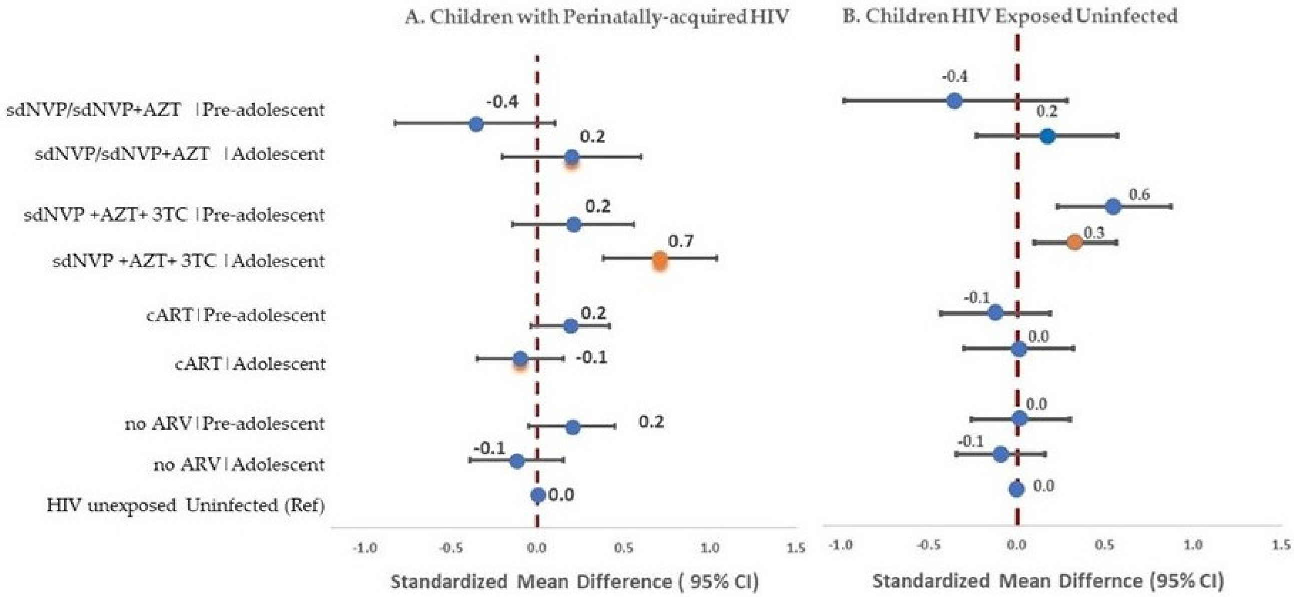

Figure S1. Peripartum-ART-exposure associated 12 months-averaged difference in functional impairment score from 6–18 years old according to HIV status and developmental stage.
